# A Comparative Study to Evaluate the Anesthetic Efficacy of Buffered Versus Non-buffered 2% Lidocaine During Inferior Alveolar Nerve Block

**DOI:** 10.7759/cureus.31855

**Published:** 2022-11-24

**Authors:** Tarang Kumar Jain, Rahul Jha, Anushree Tiwari, Neha Agrawal, Sheetal Mali, Anamika Sinha, Hiroj Bagde, Ramanpal Singh

**Affiliations:** 1 Department of Oral and Maxillofacial Surgery, Rungta College of Dental Sciences & Research, Bhilai, IND; 2 Department of Periodontology, Rungta College of Dental Sciences & Research, Bhilai, IND; 3 Department of Clinical Quality and Value, American Academy of Orthopaedic Surgeons, Rosemont, USA; 4 Department of Dentistry, Government Medical College, Mahasamund, IND; 5 Department of Conservative Dentistry and Endodontics, Bharati Vidyapeeth (Deemed to be University) Dental College and Hospital, Navi Mumbai, IND; 6 Department of Periodontology, Rama Dental College Hospital and Research Centre, Kanpur, IND; 7 Department of Oral Medicine and Radiology, New Horizon Dental College and Research Institute, Bilaspur, IND

**Keywords:** lidocaine, anesthesia, oral surgery, nerve block, efficacy

## Abstract

Background: The study aimed to compare the clinical efficacy, safety, and acceptability of buffered lidocaine (8.4% sodium bicarbonate and 2% lidocaine with 1:80,000 adrenaline) versus non-buffered lidocaine (2% lidocaine with 1:80,000 adrenaline) during inferior alveolar nerve block.

Materials and methods: Fifty patients who required bilateral extractions in a single arch were included in this study. One hundred extractions were carried out, and all of the patients had nerve blocks during the procedure. We also took note of the patient's pain level as measured on a visual analog scale, as well as the start of the action, duration of postoperative analgesia, and occurrence of any problems. The duration of anesthesia was assessed by the feeling of numbness and the first sign of pain.

Result: All the patients in both study groups reported subjective numbness of the lips and tongue. The depth of anesthesia was evaluated by pain and comfort during the procedure with a visual analog scale and showed no significant difference between the two groups. The onset of action for the pterygomandibular nerve block was 1.240.31 minutes (buffered) and 1.710.51 minutes (non-buffered). When compared, the duration of anesthesia was 327.18102.98 minutes (buffered) and 129.0826.85 minutes (non-buffered).

Conclusion: This study concludes that the buffered solution has a faster onset of action than the non-buffered solution. Both solutions exhibit similar intraoperative efficacy. The duration of postoperative anesthesia was prolonged with buffering. Buffering also reduced pain scores during the early postoperative period.

## Introduction

The use of local anesthetics in dental surgery was the standard practice even at the start of the 20th century. The inferior alveolar nerve block is often used for numbing the patient prior to mandibular reconstruction surgery. Nonetheless, pulpal numbness is not usually the consequence of blocking the inferior alveolar nerve. The success rate of inferior alveolar nerve blocks ranges from around 7% to 75%, as revealed by several experimental trials [1-3]. A basic description of pain would be an unpleasant emotional experience triggered by noxious stimuli and sent to the central nervous system through a separate neural network [2].

The genesis and conduction of nerve impulses as well as their propagation along the nerve are slowed by local anesthetics because they reduce the permeability of the neuron membrane to sodium ions [3]. Given the addictive nature of cocaine, the discovery of procaine at the turn of the 20th century led to its substitution. Mepivacaine, bupivacaine, lignocaine, and articaine are only a few of the derivatives of lidocaine that are often utilized for pain management in oral surgical operations today [4].

Adding the vasopressor epinephrine to a local anesthetic like lidocaine or a sedative like fentanyl slows the onset of the anesthetic's effects by reducing blood flow to the area and spreading the drug beyond the injection site. To prolong the vasopressor's useful life, a sedative arrangement must be prepared at a pH of around 3.5, which is low, resulting in an acidic medium that may cause irritation and discomfort at the site of infusion in certain patients. The cartridge contains a balanced mixture of RN (the uncharged, deionized, "dynamic" free base type of the drug that is lipid solvent) and RNH+ ("charged" or ionized cationic structure that does not dissolve in lipids), with the former being able to traverse the nerve film while the latter cannot.

Using the Henderson-Hasselbalch condition, we know that the total amount of constantly ionized local anesthetic in a dental cartridge is always dependent on the pH of the setup. At a pH of 3.5, for example, 99.99% of the lidocaine hydrochloride will be in the ionized structure (RNH+), which is insoluble in lipid solvents, and just 0.004% will be in the deionized form, which is soluble in lipid solvents (RN). As was previously mentioned, the ionized, nonpolar structure of a lipid solvent is the only one capable of penetrating the nerve layer. Once within the nerve, the RN picks up H+, and the subsequent RNH+ enters a sodium ion (Na+) channel to stymie nerve conduction. Until the body brings the pH of the cocktail into a more physiological range, the sedative will not take action (between 7.35 and 7.45) [5]. By raising the pH of the anesthetic solution, more of the anesthetic molecules will be in an uncharged state like RN negative ions, allowing them to enter nerve cells and produce anesthesia [6].

When there is contamination, the local pH drops, making it difficult for the standard sedative fluid to deliver effective and long-lasting pulpal drowsiness. Contaminated tissue is more acidic, increasing the difficulty of the RN change [5]. Most clinical experts believe that the sedative arrangement should cross the blood-nerve barrier and block the voltage-gated sodium channel to prevent depolarization of the nerve. Therefore, infusing the solution at a neutral pH reduces the need for and time delay associated with buffering by the tissue fluids, allowing for the fastest possible availability of the most potent unionized form of the medicine without losing its optimal nature as a vasopressor [7]. Concerning the next issue, that of injection pain, it is generally agreed that three factors are primarily to blame: pain from the needle, swelling from the anesthetic, and, most critically, the acidity of the anesthetic solution itself, when it is deposited in the tissues, all of which contribute to the unpleasantness of getting a shot [6].

Evidence for buffering's efficacy is growing; however, it is still infrequently employed in dental infusions. This is because vasoconstrictors, such as epinephrine, are unstable under increased pH, and the most popular approach of buffering adjacent sedatives must be carried out immediately before the infusion to produce the optimum benefits and ensure the safety of the vasoconstrictor. The current review intends to compare the efficacy of supported versus non-cushioned nearby sedatives during second-rate alveolar nerve block because only very limited studies have been carried out in inferior alveolar nerve block clinic settings, with the use of sodium bicarbonate-cradled sedative arrangements.

## Materials and methods

Fifty patients reporting to the Department of Oral and Maxillofacial Surgery, seeking extraction of mandibular molar teeth bilaterally and satisfying the inclusion criteria, were taken up for the study. Informed consent was taken, and the Institutional Review Board of Rungta Dental College, Bhilai, India, gave approval (RCDSR/IEC/MDS/2016/8).

Data Collection

A total of 100 cases were taken up for the study between the age groups of 18 and 60 years. Proper case history and hematological and radiographic examination were performed for each patient before the procedure. Patients aged 18-60 years who gave their informed written consent were included in the study. Patients included were advised to have bilateral multiple mandibular posterior teeth extracted. Women who were either pregnant or breastfeeding and patients with severe allergies to local anesthetics, a history of severe medical conditions, a weakened immune system, mental illness, or a physical disability were excluded from the study. The researchers intended to conduct a split-mouth clinical trial. Two groups, each having 50 patients, were formed: Group A, which did not receive a buffer, received 2% lidocaine and 1:180,000 adrenaline, and Group B used a buffer that included 2% lidocaine, 1:80,000 adrenaline, and 8.4% sodium bicarbonate. An individual was responsible for administering the local anesthetic. The equipment was fine-tuned and reinforced by an expert in oral and maxillofacial surgery. All of the participants were randomly placed into either Group A or Group B.

*Buffered Local Anesthetic Agent Preparation* 

A 1.8 ml injection solution of 2% lidocaine with 1:80,000 epinephrine was diluted with 0.18 ml of the 8.4% sodium bicarbonate solution.

Surgical Technique

After a thorough case history and clinical or radiographic evaluation, a patient was enrolled in the research. Any patient who agreed to take part in the study was randomly placed in one of two groups. Prior to the treatment, patients were instructed to gargle with chlorhexidine gluconate mouthwash for 10 minutes. SODAC (8.4% sodium bicarbonate) from Neon Laboratories Ltd. (Mumbai, India) and 2% lidocaine diluted with adrenaline at a 1:80,000 ratio were utilized. The inferior alveolar nerve block used 1.5 ml of the solution placed using the traditional method, whereas the lingual nerve block used 0.3 ml. Additional anesthetic solutions were available for reinjection if required during the procedure. The clinical parameters were recorded, and extraction was performed. Once the tooth was removed, the socket was irrigated with normal saline. Hemostasis was achieved. Post-extraction instructions were given, and the patient was recalled for follow-up. The standard treatment for all patients consisted of 500 mg of amoxicillin every eight hours for five days postoperative to reduce the chances of secondary infection, along with 400 mg of ibuprofen every eight hours for discomfort. In the event of an allergy to amoxicillin, erythromycin 500 mg was administered eight times per day over the course of five days.

A 100-mm visual analog scale (VAS) was used to quantify patients' reports of subjective pain, with zero indicating no discomfort and 100 indicating the worst agony imaginable. The amount of anesthetic given in milliliters was checked. When an anesthetic has taken effect, the patient will no longer feel pain in their lower lip, tongue, or mucosa (in minutes). The surgeon used a three-point scale to rate how well the local anesthetic kept the patient from feeling pain during the extraction: 

A. A majority of patients in Group B, including women, felt no pain throughout the surgery.

B. Only a minority of patients in Group B felt pain throughout the process.

C. The patients who felt pain during the process were administered further anesthetic agents.

After anesthesia was given, the time taken to complete the extraction was noted. The time at which the first analgesic drug was administered after surgery was used to calculate the total period of pain relief (in minutes). The insensitivity of the mucosa, tongue, and lower lip after surgery was a visual representation of how long the soft tissues were under anesthesia. All sensations in the soft tissues were timestamped at the instant they reverted to normal. The treating physician or patient recorded the occurrence, categorization, and degree of problems (e.g., nervousness, dizziness, tremors, blurred vision, or any indication of effects on the cardiovascular and central nervous systems).

The data were analyzed using inferential and descriptive statistical methods. Descriptive statistics were used for just that: giving a bird's-eye perspective of a dataset. Chi-square and Student's unpaired *t*-test statistics were used to examine the data, make conclusions, and offer descriptive analysis. IBM SPSS Statistics for Windows, Version 22.0 (Released 2013; IBM Corp; Armonk, New York, United States) and GraphPad Prism 6.0, Released August 2012 (Dotmatics, Boston, Massachusetts) were used for the statistical analysis. In most cases, the level of statistical significance is determined by the presence or absence of a *p*-value of 0.05.

## Results

The average age of Group A participants was 42.70 ± 12.56 years and that of Group B participants was 42.04 ± 12.76 years. This study was conducted with an equal number of male and female volunteers of all ages (*p* > 0.99 for both genders).

Using a VAS, we found that the pain experienced by the non-buffered group (2% lidocaine with 1:80,000 adrenaline) during injection was 3.54, whereas the pain reported by the buffered group was only 0.96 (Table 1).

**Table 1:** Comparison of pain at the time of injection in the two groups.

Group	N	Mean	Standard deviation	Standard error mean	*p*-value
Non-buffered	50	3.54	1.78	0.25	0.0001
Buffered	50	0.96	0.80	0.11	

The result was statistically significant with *p-*value of 0.0001. When compared to patients receiving the unbuffered lignocaine therapy, those receiving the buffered lignocaine treatment reported much less pain. Patients in the unsupported group used an average of 1.81 ml of narcotic preparation (1.80% adrenaline in 2% lidocaine), whereas those in the supported group used an average of 1.66 ml of narcotic preparation (18% sodium bicarbonate in 2% lidocaine). The Student's *t*-test was conducted and found that there is a statistically significant difference (*p *= 0.0001) between the two groups regarding the total amount of narcotic medicine used by their patients (Table 2).

**Table 2:** Comparison of total volume of anesthetic agent used (ml) in the two groups.

Group	*N*	Mean	Standard deviation	Standard error mean	*p*-value
Non-buffered	50	1.81	0.21	0.03	0.0001
Buffered	50	1.66	0.17	0.02	

The mucosa, the lower lip, and the corresponding portion of the tongue were tested for sensitivity. For the buffered group, the onset of anesthesia was at 1.24 minutes, whereas it took 1.71 minutes for the non-buffered group. Both solutions include 2% lidocaine; however, the buffered solution (with 8.4% sodium bicarbonate and 2% lidocaine) begins functioning more quickly because of the sodium bicarbonate (1:80,000) (*p *= 0.0001) (Table 3).

**Table 3:** Comparison of onset of action of anesthetic agent in the two groups.

Group	N	Mean	Standard deviation	Standard error mean	*p*-value
Non-buffered	50	1.71	0.51	0.07	0.0001
Buffered	50	1.24	0.31	0.04	

The duration of analgesia was calculated as the time elapsed between when surgery was completed and when the first analgesic drug was administered. For postoperative pain relief, the buffered group had relief for 327.18 minutes on average, whereas the non-buffered group experienced relief for 129.08 minutes on average. As a result, the duration of postoperative analgesia was considerably longer in the buffered group than in the non-buffered group (*p *= 0.0001) (Table 4).

**Table 4:** Comparison of duration of postoperative anesthesia in the two groups.

Group	N	Mean	Standard deviation	Standard error mean	*p*-value
Non-buffered	50	129.08	26.85	3.79	0.0001
Buffered	50	327.18	102.98	14.56	

The mean pain on VAS in the non-buffered group and the buffered group was 32.60 ± 14.25 and 5.40 ± 5.42, respectively. By using Student’s unpaired *t*-test, a statistically significant difference was found in the mean pain on VAS in the two groups (*p* = 0.0001) (Table 5).

**Table 5:** Comparison of pain on VAS in the two groups. VAS: visual analog scale

Group	N	Mean	Standard deviation	Standard error nean	*p*-value
Non-buffered	50	32.60	14.25	2.01	0.0001
Buffered	50	5.40	5.42	0.76	

At one hour, 100% of the patients in the non-buffered group had no pain; 20% of the patients at three hours had no pain, 40% had mild pain, 28% had moderate pain, and 12% had severe pain; 2% of the patients at eight hours had no pain, 18% had mild pain, 26% had moderate pain, and 54% had severe pain. A statistically significant difference in pain on VAS was found in the non-buffered group at one hour, three hours, and eight hours using the chi-square test (*p *= 0.000).

At one hour, 100% of the patients in the buffered group had no pain; 98% of the patients at three hours had no pain and 2% had mild pain; 36% of the patients at eight hours had no pain, 50% had mild pain, 10% had moderate pain, and 4% had severe pain. A statistically significant difference in pain on VAS was found in the buffered group at one hour, three hours, and eight hours using the chi-square test (two-value = 71.218, *p *= 0.0001, S). Using the chi-square test, a statistically significant difference in pain on VAS was found in the two groups at three hours (two-value = 62.97, *p* = 0.0001, S) and eight hours (two-value = 47.85, *p* = 0.0001, S) (Table 6).

**Table 6:** Comparison of pain on VAS in the two groups when anesthesia was done. VAS: visual analog scale

Pain on VAS	Non-buffered			Buffered		
	One hour	Three hours	Eight hours	One hour	Three hours	Eight hours
No pain	50 (100%)	10 (20%)	1 (2%)	50 (100%)	49 (98%)	18 (36%)
Mild pain	0 (0%)	20 (40%)	9 (18%)	0 (0%)	1 (2%)	25 (50%)
Moderate pain	0 (0%)	14 (28%)	13 (26%)	0 (0%)	0 (0%)	5 (10%)
Severe pain	0 (0%)	6 (12%)	27 (54%)	0 (0%)	0 (0%)	2 (4%)
Total	50 (100%)	50 (100%)	50 (100%)	50 (100%)	50 (100%)	50 (100%)
*p*-value (in two groups)				-	0.0001	0.0001
*p*-value (within group)	0.0001			0.0001		

## Discussion

It is vital to thoroughly numb the region before extraction in order to secure patient participation and control the patient's anxiety throughout the procedure. The patient's pain tolerance and the effectiveness of the local anesthetic both have a role in how much discomfort they experience [8]. Local anesthetic administration may take several forms, each tailored to meet the needs of a particular clinical scenario. In oral surgery, lignocaine is a frequent local anesthetic. It has a rapid start, lasts long enough, and has little adverse effects since it comes from the amide family [9].

The combination of the buffering action of bicarbonate and the production of carbon dioxide means that the pain on injection and onset time are reduced, with the provision of a better depth of anesthesia leading to increased patient comfort. However, there is no adequate experimental proof showing an alteration in the duration of anesthesia due to buffering. To get our hands on some buffered lidocaine, we might do it in two different methods. The first and most practical is to make your own lidocaine buffer using a solution like 8.4% sodium bicarbonate, which can be purchased for less than Canadian dollar (CAD) 5 a bottle and is widely available. Before injection, just combine the two in a syringe or container. The volume of liquid that can be stored in a 10 ml syringe is 11 ml, whereas a 20 ml syringe can contain 22 ml. In order to get the desired 1:10 ratio, a 10 ml syringe is filled to 11 ml with 1 ml of bicarbonate and 10 ml of 1% lidocaine with 1:100,000 epinephrine [10]. The success rates for administering anesthesia through inferior alveolar nerve block were comparable between the present study and previous research on this technique [11]. Due to the acid dissociation constant (pKa) of lidocaine being 7.9, we considered formulating an anesthetic with a pH of 7.9, resulting in an equal amount of the cation and base form [12,13].

The onset timings of the various anesthetic preparations were not significantly different. Researchers observed that anesthetic formulations with higher pH values had a quicker onset time. This was the case in studies by DiFazio et al. [14], Zahl et al. [15], Benzon et al. [16], and Sinnott et al. [13]. However, neither DiFazio et al. [14] nor Christoph et al. [17] found that drugs with their pH altered had a quicker onset. It seems to reason that there would be more of the uncharged base accessible in the higher pH-adjusted anesthetic formulation at the outset. For that reason, we could expect a quicker onset. While the lidocaine-sodium bicarbonate combination seemed promising, we were unable to find statistical evidence that it really worked quicker. It was shown by Nusstein et al. in a retrospective analysis of inferior alveolar nerve blocks using 2% lidocaine and 1:80,000 epinephrine that a higher percentage of patients had moderate-to-severe pain immediately after needle insertion [18]. After needle insertion, 20-22% of people reported moderate-to-severe pain, and there was no statistically significant difference between the anesthetic formulations even when 0.2 ml of anesthetic solution was deposited beforehand. Research by Nusstein et al. found that 22-56% of participants had moderate-to-severe pain after needle insertion into the treatment area [19]. The incidence of mild-to-severe pain due to solution deposition did not vary significantly (2-8%) among the various anesthetic preparations. A buffered anesthetic solution containing sodium bicarbonate significantly decreased the pain of injection in research involving intradermal injections conducted by Christoph et al. [17] and McKay et al. [20]. Contrarily, studies by Gershon et al. [21] and Burns et al. [22] found that intradermal anesthesia using buffered anesthetic solutions did not appreciably lessen pain. Because we used an inferior alveolar nerve block, it is possible that our results will not be applicable to other studies. The study by Bunke et al. indicated that adding sodium bicarbonate to a lidocaine solution did not substantially lessen injection pain [23]. 

## Conclusions

In this study, buffered lignocaine caused less injection discomfort than standard lignocaine. The action started faster and was more immersive. Both remedies had varying durations of impact. Injecting a buffered local anesthetic with lidocaine and epinephrine decreases injection site discomfort while having the same anesthetic activity and duration as a more concentrated unbuffered solution. Due to reduced injection discomfort, these preparations may be used for five-hour surgeries. Buffered lignocaine reduces injection and procedure discomfort, making dental and oral surgical operations under local anesthesia more comfortable for patients. However, multicentric research with larger sample sizes could increase buffered lignocaine's shelf life and production cost, making it a valuable tool in oral and maxillofacial surgery.
